# Clinical outcomes and risk factors associated with bloodstream infections caused by multidrug-resistant Gram-negative bacteria in hospitalized patients: a prospective cohort study in Libya

**DOI:** 10.1099/jmm.0.002101

**Published:** 2025-11-27

**Authors:** Mohanned Mohamed Alwashaish

**Affiliations:** 1 Microbiology Department, Faculty of Pharmacy, Misurata University, Misurata, Libya

**Keywords:** antimicrobial stewardship, bloodstream infection (BSI), Gram-negative bacteria, Libya, mortality, multidrug resistance (MDR)

## Abstract

**Introduction.** Bloodstream infections (BSIs) caused by multidrug-resistant Gram-negative bacteria (MDR-GNB) are a major cause of mortality, with limited treatment options.

**Gap/Hypothesis.** Despite the global rise of MDR-GNB, prospective data on their clinical impact in North Africa remain scarce, restricting evidence-based guidance for empiric therapy.

**Aim.** To assess the prevalence, resistance patterns and clinical outcomes of MDR-GNB BSIs in Libya, and to identify predictors of 30-day mortality.

**Methodology.** A prospective cohort study was conducted in two Libyan hospitals between November 2022 and November 2024. Adult patients with positive blood cultures were enrolled. Isolates were identified using standard microbiological methods, and antimicrobial susceptibility testing was interpreted according to Clinical and Laboratory Standards Institute (CLSI) 2023 guidelines. Multivariable logistic regression was applied to identify independent predictors of 30-day mortality.

**Results.** Among 673 BSI episodes, 37.4% were multidrug resistance (MDR). *Escherichia coli* (32.0%) and *Klebsiella pneumoniae* (25.9%) predominated, while carbapenem resistance was highest in *Acinetobacter baumannii* (42.4%). Overall, 30-day mortality was 23.8%, and was significantly higher in MDR infections (32.1% vs 18.8%; *P*<0.001). Independent predictors of mortality were MDR infection [adjusted odds ratio (aOR), 1.9], Intensive care Unit (ICU) admission (aOR, 2.6), Charlson Comorbidity Index ≥3 (aOR, 1.7) and inappropriate empiric therapy (aOR, 2.3).

**Conclusion.** MDR-GNB BSIs are highly prevalent in Libya and substantially worsen outcomes. These findings highlight the urgent need for improved empiric therapy, antimicrobial stewardship and infection control programmes.

## Data Summary

All data generated or analysed during this study are included in this article.

## Introduction

Bloodstream infections (BSIs) are a leading cause of morbidity and mortality worldwide, increasingly complicated by multidrug-resistant Gram-negative bacteria (MDR-GNB). These pathogens delay effective therapy and are associated with longer hospitalization and higher mortality [1,3]. Recent global data, such as the European prospective cohort study on bloodstream infections caused by multidrug-resistant Gram-negative bacteria (EUROBACT-2) cohort, identify Gram-negative organisms as the main cause of hospital-acquired BSIs and link carbapenem resistance with poor outcomes [1]. The burden is greatest in low- and middle-income countries, where limited access to advanced antimicrobials and weak infection control aggravate the crisis [4,6]. In North Africa, and particularly in Libya, clinical data on MDR-GNB BSIs remain scarce, with most reports limited to resistance rates rather than patient outcomes [7]. This prospective study (2022–2024) examined BSIs in two Libyan tertiary hospitals, assessing MDR-GNB prevalence, resistance profiles and clinical outcomes, including 30-day mortality and adequacy of empiric therapy. The findings provide essential evidence to guide empiric treatment and strengthen antimicrobial stewardship in resource-limited settings.

## Methods

### Study design and setting

This prospective cohort study followed Strengthening the Reporting of Observational Studies in Epidemiology (STROBE) guidelines and was conducted at Misurata Medical Center and the National Institute for Cancer Treatment, Libya, between November 2022 and November 2024. Misurata Medical Center (≈140 beds, including 25 Intensive Care Unit (ICU) beds) and the National Institute for Cancer Treatment (≈120 beds, including 20 ICU beds) are tertiary-care facilities with in-house microbiology laboratories performing culture, bacterial identification and antimicrobial susceptibility testing. Molecular assays (e.g. PCR for carbapenemase genes) were unavailable during the study period.

### Participants

**Fig. 1.**
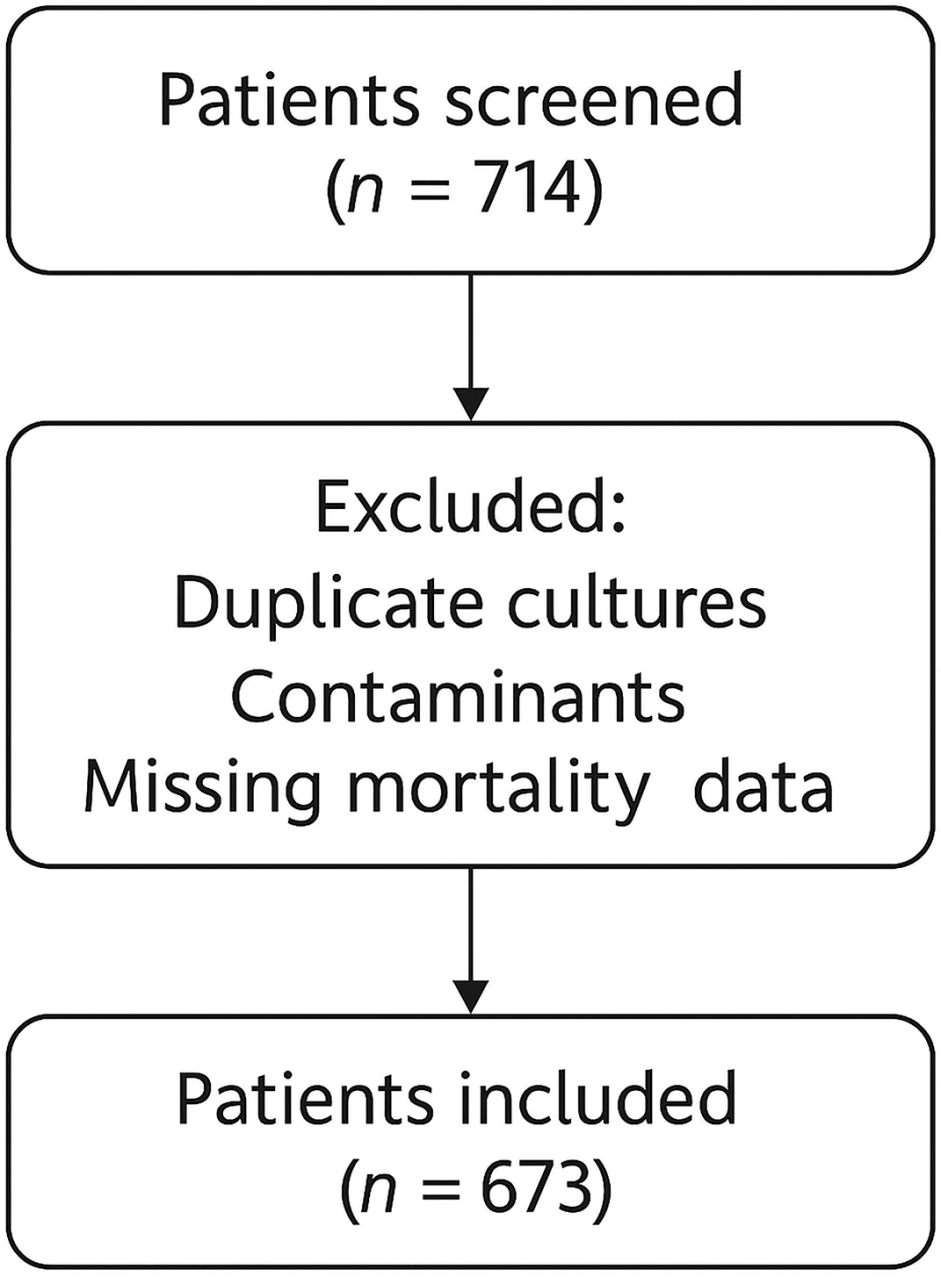
Flow diagram summarizing patient screening and inclusion for the study cohort.

Adults (≥18 years) with at least one positive blood culture yielding a recognized bacterial pathogen were eligible. Only the first episode per patient was included. Repeat isolates within 14 days, contaminants per standard criteria and cases lacking 30-day mortality data were excluded. Fungal BSIs were omitted. A total of 714 patients were screened; after exclusions, 673 were included in the final analysis (Fig. 1).

### Microbiological procedures

Blood cultures (two bottles: aerobic + anaerobic, 8–10 ml each) were obtained aseptically and incubated in automated systems. Identification used standard biochemical tests, API 20E/NE strips and matrix-assisted laser desorption/ionization-time of flight (MALDI-TOF) mass spectrometry when available. Susceptibility testing employed the Kirby–Bauer disc diffusion method and Clinical and Laboratory Standards Institute (CLSI) 2023 interpretations [8]. Extended-Spectrum β-Lactamase (ESBL) production was confirmed by the double-disc synergy method [9,10], and carbapenemase activity by Modified and EDTA-Modified Carbapenem Inactivation Methods (mCIM/eCIM) or Carba NP [11]. Multidrug resistance (MDR) was defined as non-susceptibility to ≥3 antimicrobial classes [12]. Both laboratories implemented CLSI-recommended Quality Control (QC) using daily verification of media and discs. The contamination rate was <3%.

### Clinical definitions

Appropriate empiric therapy was defined as administration of at least one active antimicrobial within 24 h of blood culture collection [13]. The primary outcome was 30-day all-cause mortality, with hospital length of stay and 30-day readmission as secondary outcomes. Planned collection of antibiotic cost data was not completed due to incomplete pharmacy records and was therefore excluded from analysis.

### Data collection

Demographic, clinical and therapeutic variables were recorded using standardized case report forms. Comorbidities were quantified using the Charlson Comorbidity Index [14]. Data were double-checked for accuracy, and potential contaminants were independently reviewed by two infectious disease specialists. Prior antibiotic exposure was defined as receipt of systemic antimicrobial therapy within 90 days before the index culture, including β-lactams (penicillins, cephalosporins, carbapenems), fluoroquinolones, aminoglycosides or colistin.

### Sample size

All eligible cases during the 24-month study period were included (*n*=673). This sample size provided >80% statistical power to detect an odds ratio ≥1.7 for mortality associated with MDR status at α=0.05.

### Statistical analysis

Categorical variables were analysed with χ² or Fisher’s exact tests, and continuous variables with t-test or Mann–Whitney U test, as appropriate. Survival was assessed using Kaplan–Meier curves and the log-rank test. Independent predictors of 30-day mortality were determined by multivariable logistic regression, adjusted for demographic and clinical factors, with results reported as adjusted odds ratios (aORs) and 95% confidence intervals (CIs). All analyses were performed using SPSS v25 and R v4.2.2.

### Ethical considerations

The study protocol was reviewed and approved by the Faculty of Pharmacy, Misurata University (Approval No. 79/2022), and by the institutional review boards of both participating hospitals. As only anonymized data were used, the requirement for informed consent was waived.

## Results

### Patient characteristics

During the 24-month study period, 673 patients with BSIs were enrolled. The median age was 56 years (Interquartile Range (IQR), 42–68), and 58% were. Compared with non-MDR cases, patients with MDR infections were older (median, 59 vs 54 years; *P*=0.04), more frequently admitted to the ICU (43.3% vs 25.6%; *P*<0.001) and more likely to have diabetes (40.9% vs 29.9%; *P*=0.002) or prior antibiotic exposure within 90 days (54.0% vs 39.9%; *P*<0.001). Among those with prior exposure, the most commonly used antibiotic classes were β-lactams (64%), fluoroquinolones (22%) and aminoglycosides (10%). No significant differences were observed for sex or chronic kidney disease (Table 1).

**Table 1.** Baseline characteristics of patients with BSIs

Characteristic	Overall (*n*=673)	Non-MDR (*n*=421)	MDR (*n*=252)	*P*-value
Age, median (IQR), years	56 (42–68)	54 (40–66)	59 (46–70)	0.04*
Male sex, *n* (%)	391 (58.1)	236 (56.0)	155 (61.5)	0.16†
ICU at culture, *n* (%)	217 (32.2)	108 (25.6)	109 (43.3)	na
Diabetes mellitus, *n* (%)	229 (34.0)	126 (29.9)	103 (40.9)	0.002†
CKD, *n* (%)	129 (19.2)	72 (17.1)	57 (22.6)	0.07†
Prior antibiotics (90 d), *n* (%)	304 (45.2)	168 (39.9)	136 (54.0)	na

*p<0.05.

†p<0.01.

CKD, chronic kidney disease; IQR, Interquartile range; na, not applicable.

### Microbiological findings

**Fig. 2.**
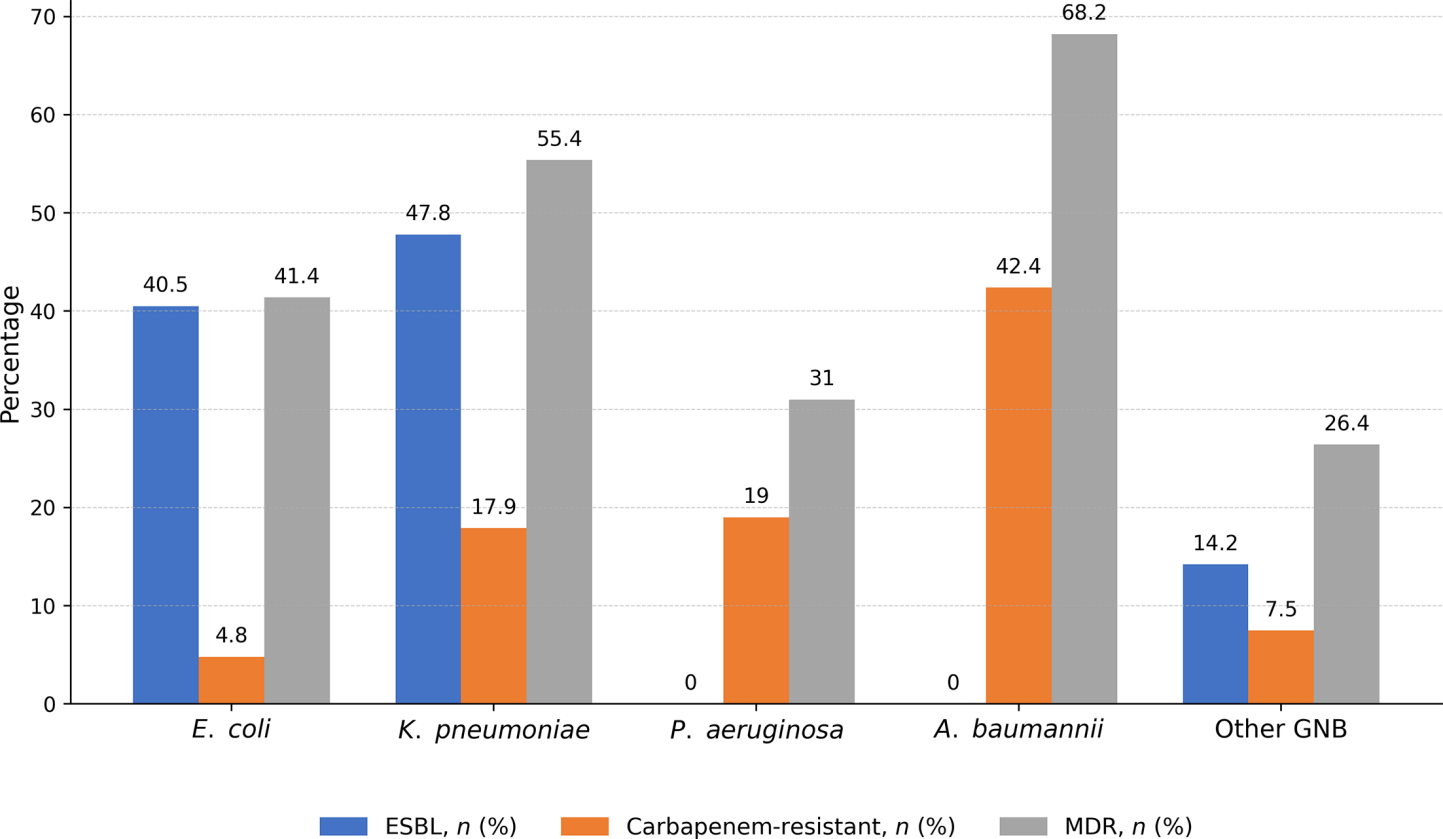
Resistance profiles of major Gram-negative pathogens. Percentages reflect the proportion of isolates exhibiting extended-spectrum β-lactamase (ESBL) production, carbapenem resistance, and multidrug resistance MDR. Values above the bars indicate the percentage of isolates within each species group.

A total of 709 Gram-negative isolates were recovered. *Escherichia coli* (32.0%), *Klebsiella pneumoniae* (25.9%) and *Pseudomonas aeruginosa* (17.8%) were predominant. Among Enterobacterales, 42% were ESBL producing. Carbapenem resistance was most frequent in *Acinetobacter baumannii* (42.4%) and *K. pneumoniae* (17.9%), but remained uncommon in *E. coli* (4.8%). Overall, 37.4% of infections were MDR. Amikacin and colistin retained the highest activity (>85% susceptibility), while resistance to third-generation cephalosporins exceeded 45% in Enterobacterales (Table 2, Fig. 2).

**Table 2.** Distribution of major pathogens and antimicrobial resistance profiles

Pathogen (*n*=709)	ESBL, *n* (%)	Carbapenem resistant, *n* (%)	MDR, *n* (%)
*E. coli* (227)	92 (40.5)	11 (4.8)	94 (41.4)
*K. pneumoniae* (184)	88 (47.8)	33 (17.9)	102 (55.4)
*P. aeruginosa* (126)	nr	24 (19.0)	39 (31.0)
*A. baumannii* (66)	nr	28 (42.4)	45 (68.2)
Other GNB (106)	15 (14.2)	8 (7.5)	28 (26.4)

MDR was defined as non-susceptibility to ≥3 antimicrobial classes.

The number of ESBL-producing isolates does not exactly correspond to the total number resistant to third-generation cephalosporins, as some non-ESBL strains exhibited alternative resistance mechanisms leading to cephalosporin resistance.

nr, no results detected.

### Antibiotic resistance pattern

**Fig. 3.**
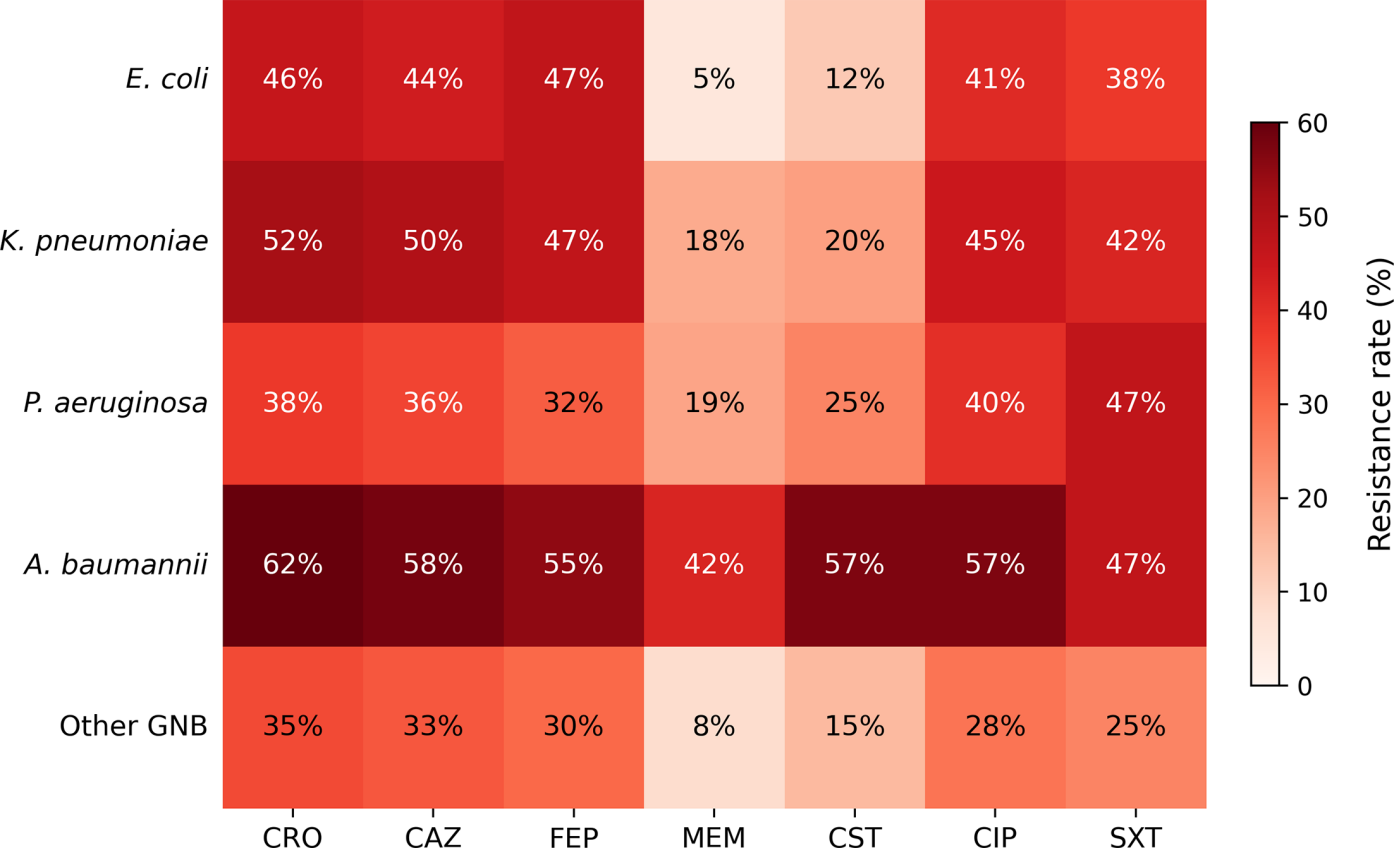
Heatmap of antimicrobial resistance rates (%) among major Gram-negative bloodstream pathogens. Resistance to tested antibiotics is displayed for Gram-negative bacilli. Percentages were calculated per pathogen group, and values are shown within each cell. Significance: The deeper the red color, the higher the resistance rate (%) for that organism–antibiotic combination.

Resistance to third-generation cephalosporins surpassed 40% in *E. coli* and 50% in *K. pneumoniae*, consistent with high ESBL prevalence. Carbapenem resistance was highest in *A. baumannii* (42.4%), followed by *K. pneumoniae* (17.9%). Amikacin preserved good activity against Enterobacterales (>80%), but resistance exceeded 25% in *P. aeruginosa* and 34.8% in * A. baumannii*. Colistin retained broad activity (>90% susceptible), although resistance was present in 18.2% of *A. baumannii*. Ciprofloxacin resistance was widespread across species (>40%) (Table 3, Fig. 3).

**Table 3.** Antimicrobial resistance profiles of major Gram-negative bloodstream isolates

Pathogen (*n*)	CRO R, *n* (%)	CAZ R, *n* (%)	FEP R, *n* (%)	MEM R, *n* (%)	AMK R, *n* (%)	CST R, *n* (%)	CIP R, *n* (%)	SXT R, *n* (%)
*E. coli* (227)	104 (45.8)	100 (44.1)	95 (41.9)	11 (4.8)	27 (11.9)	9 (4.0)	93 (41.0)	86 (37.9)
*K. pneumoniae* (184)	96 (52.2)	92 (50.0)	87 (47.3)	33 (17.9)	37 (20.1)	13 (7.1)	83 (45.1)	77 (41.8)
*P. aeruginosa* (126)	48 (38.1)	45 (35.7)	40 (31.7)	24 (19.0)	32 (25.4)	13 (10.3)	50 (39.7)	nr
*A. baumannii* (66)	40 (60.6)	38 (57.6)	36 (54.5)	28 (42.4)	23 (34.8)	12 (18.2)	34 (51.5)	31 (47.0)
Other GNB (106)	37 (34.9)	35 (33.0)	32 (30.2)	8 (7.5)	16 (15.1)	5 (4.7)	30 (28.3)	27 (25.5)

Comparisons were made using χ² or Fisher’s exact test, as appropriate. *A. baumannii* showed significantly higher resistance to MEM than other pathogens (*P*<0.001), while *K. pneumoniae* exhibited greater resistance to CRO than *E. coli* (*P*=0.03). Resistance to AMK remained significantly lower than resistance to β-lactams across all Enterobacterales (*P*<0.01).

AMK, amikacin; CAZ, ceftazidime; CIP, ciprofloxacin; CRO, ceftriaxone; CST, colistin; FEP, cefepime; MEM, meropenem; n, number; nr, no results detected; R, resistance; SXT, trimethoprim–sulfamethoxazole.

### Outcomes

**Fig. 4.**
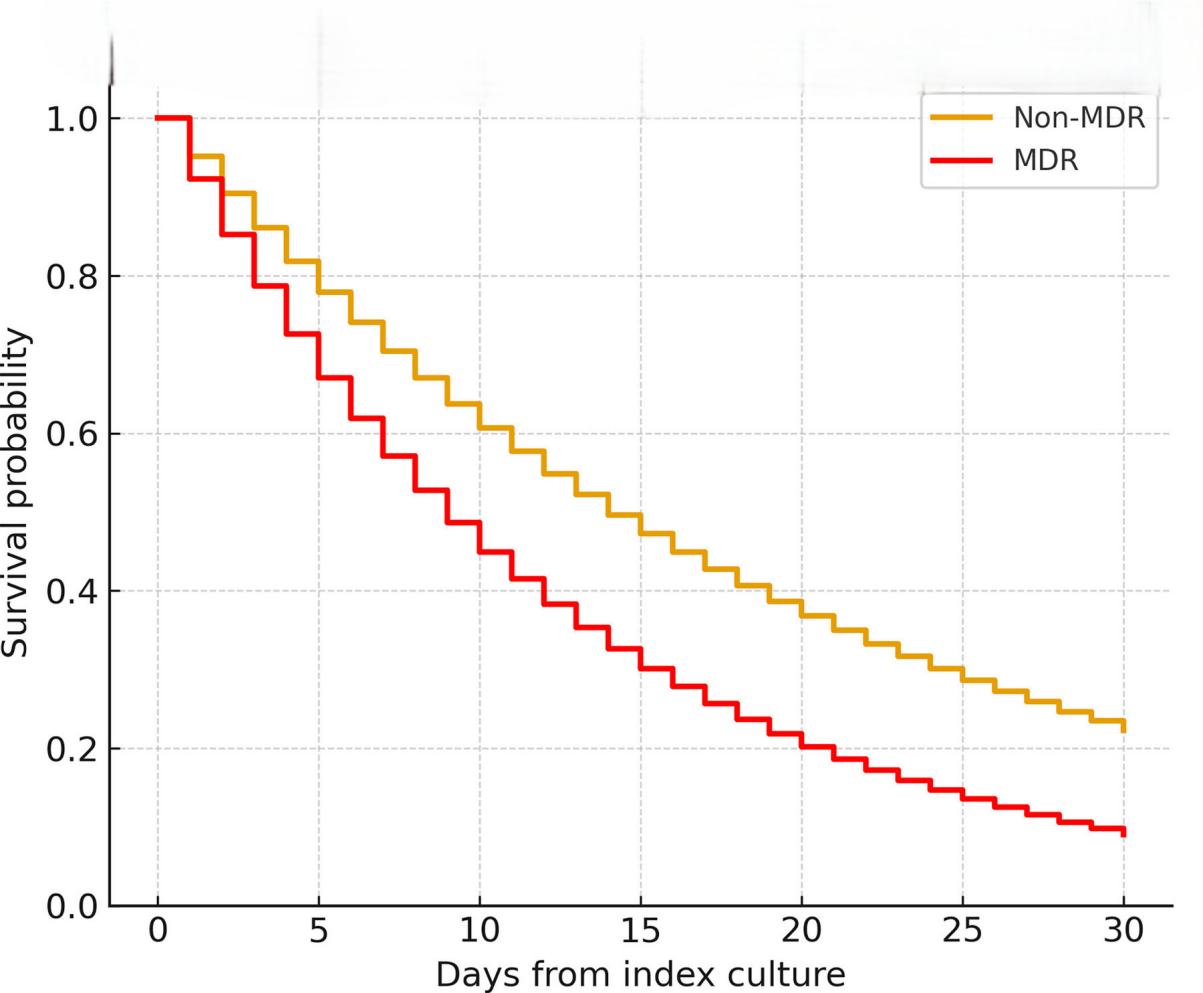
Kaplan–Meier survival curves: 30-day survival was significantly lower among MDR infections (log-rank *P*<0.001).

The overall 30-day all-cause mortality was 23.8% (160/673), and was significantly higher in MDR infections than in non-MDR infections (32.1% vs 18.8%; *P*<0.001). Kaplan–Meier analysis demonstrated reduced survival among MDR infections (log-rank *P*<0.001; Fig. 4).

On multivariable logistic regression, independent predictors of 30-day mortality were:

- MDR infection (aOR, 1.9; 95% CI: 1.3–2.8)
- ICU admission (aOR, 2.6; 95% CI: 1.8–3.9)
- Charlson Comorbidity Index ≥3 (aOR, 1.7; 95% CI: 1.2–2.5)
- Inappropriate empiric therapy (aOR, 2.3; 95% CI: 1.6–3.4).

**Fig. 5.**
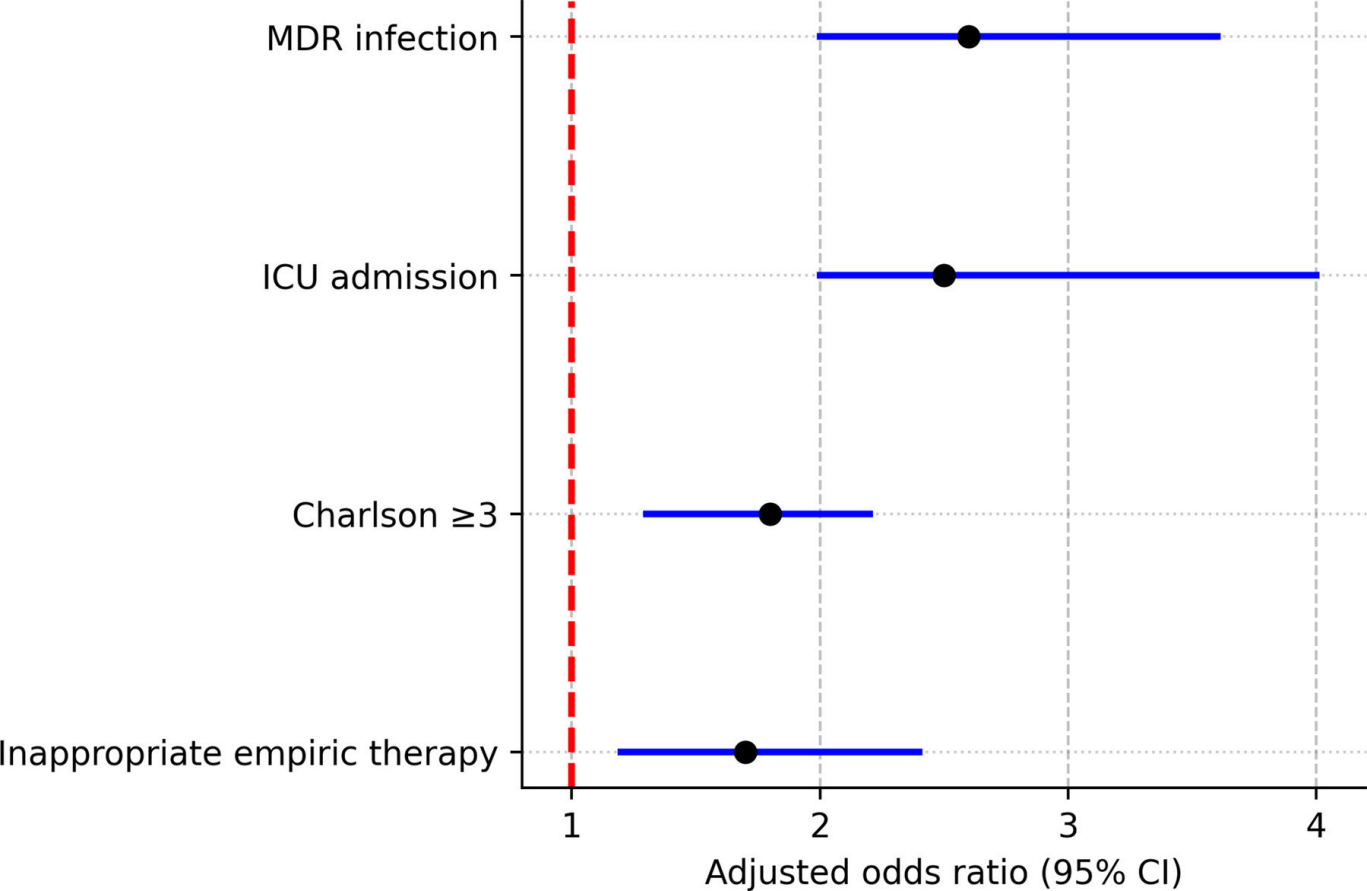
Forest plot of the multivariable logistic regression model.

Fig. 5 presented a Forest plot of the multivariable logistic regression model.

Median hospital stay was longer in MDR cases (14 vs 10 days; *P*<0.01). Inappropriate empiric therapy occurred in 41% of MDR infections compared with 14% in non-MDR infections (*P*<0.001). These associations are summarized in Fig. 5.

## Discussion

This prospective study reveals a substantial burden of BSIs caused by MDR-GNB in Libya, underscoring their serious clinical and public health impact. These findings align with reports from Saudi Arabia and Egypt, showing high MDR rates and poor outcomes in Gram-negative BSIs [15,16], as well as studies from Tunisia and Morocco highlighting ICU-associated transmission and ongoing healthcare-related spread of resistance [17,18].

### Risk factors and patient characteristics

MDR BSIs in our cohort were more common among older patients, ICU admissions, individuals with diabetes and those with prior antibiotic exposure. Diabetes is a well-recognized predisposing factor due to immune dysfunction [19,20]. Recent systematic reviews and regional cohort studies confirm that prior antibiotic exposure, high comorbidity burden and ICU stay are consistent risk factors for MDR Gram-negative BSIs [21,23]. A meta-analysis showed that inappropriate empiric therapy is strongly associated with these same host factors, emphasizing the need for risk stratification in guiding empiric therapy [24].

### Microbiological findings

*E. coli* and *K. pneumoniae* were the predominant bloodstream pathogens, consistent with global trends where urinary and intra-abdominal sources predominate [25]. Their dominance reflects gut and urinary tract colonization and their high capacity to acquire plasmid-mediated resistance determinants, enabling persistence under antibiotic pressure. The frequent isolation of *A. baumannii* and *P. aeruginosa* highlights the ICU environment, where invasive procedures, mechanical ventilation and selective antibiotic exposure favour these opportunistic organisms [26].

More than 40% of *Enterobacterales* were ESBL producers, aligning with global increases in Cefotaximase-Munich (CTX-M) type enzymes [27] and regional reports of widespread ESBL-producing *K. pneumoniae* in North Africa [28]. This high prevalence likely results from extensive use of cephalosporins and fluoroquinolones without adequate antimicrobial stewardship oversight, promoting horizontal spread of ESBL genes.

Carbapenem resistance reached 42% in *A. baumannii* and 18% in *K. pneumoniae*, consistent with global surveillance data [29,30]. In *K. pneumoniae*, resistance is primarily plasmid mediated through New Delhi Metallo-β-lactamase (NDM) and Oxacillinase-48 (OXA-48) like carbapenemases, while *A. baumannii* exhibits combined mechanisms involving OXA-type enzymes, efflux systems and porin loss [31]. These mechanisms critically limit therapeutic options and elevate the risk of hospital outbreaks.

### Resistance patterns

*P. aeruginosa* was a major contributor to MDR BSIs. Its persistence in hospital water systems, biofilm formation and disinfectant tolerance explain its dominance in ICU outbreaks [32]. Global surveillance initiatives World Health Organization Global Antimicrobial Resistance Surveillance System(WHO GLASS), European Centre for Disease Prevention and Control (ECDC) classify these organisms as high-priority threats, especially in resource-limited regions [33,34].

Resistance to third-generation cephalosporins was widespread, consistent with global dissemination of CTX-M-type ESBLs [35,36], undermining the use of ceftriaxone and ceftazidime for empiric therapy. Carbapenem resistance among *K. pneumoniae* and *A. baumannii* aligns with the WHO’s ‘critical-priority’ category demanding urgent Research and Development (R and D) investment [37]. The continued spread of carbapenemase-producing *Enterobacteriaceae* and carbapenem-resistant *A. baumannii* highlights the risk of cross-border outbreaks if containment is weak [38,40].

Aminoglycosides, particularly amikacin, retained the highest activity, consistent with prior reports of preserved efficacy against ESBL producers [41]. Their limited use, compared with β-lactams, may explain the lower resistance levels. However, non-fermenters displayed higher resistance, mainly due to aminoglycoside-modifying enzymes and efflux mechanisms [42]. Colistin remained active against most isolates, though its reliability is compromised by testing variability and the emergence of plasmid-mediated *mcr-1* resistance [43,44]. Thus, colistin remains a salvage option rather than a sustainable solution.

Fluoroquinolone resistance exceeded 40–50%, reflecting widespread clinical and community use and driven by both chromosomal mutations (*gyrA*, *parC*) and plasmid-mediated determinants [45]. The overall resistance landscape underscores the urgent need for local antibiogram-guided empiric therapy and strengthened stewardship to preserve remaining active agents.

### Clinical outcomes

The overall 30-day mortality increased among MDR infections. MDR status independently predicted death, consistent with international evidence linking resistance to delays in appropriate therapy and poorer survival [46]. Inappropriate empiric therapy was also strongly associated with mortality, echoing findings from major sepsis cohorts [47]. The biological rationale is clear: delayed active therapy allows bacterial proliferation and systemic inflammation, progressing to organ failure.

ICU admission further predicted death, reflecting both disease severity and increased MDR exposure in critical-care settings [48]. Likewise, higher Charlson Comorbidity Index scores were associated with excess mortality, consistent with impaired host defences and reduced resilience to septic shock [49]. Regional studies from Saudi Arabia and Tunisia report similar patterns, associating MDR BSIs with prolonged hospitalization, greater costs and higher death rates [50,51]. Globally, inappropriate empiric therapy remains a leading preventable driver of sepsis-related mortality [52,53].

### Clinical and public health implications

The high prevalence of ESBL- and carbapenem-resistant pathogens compromises empiric therapy, increases preventable deaths and strains healthcare systems in low- and middle-income settings [54,56]. Tailored empiric regimens based on local resistance profiles, restricted carbapenem use and aminoglycoside adjuncts may improve outcomes. Strengthening rapid diagnostics, antimicrobial stewardship and infection control aligned with the WHO Global Action Plan is essential [57,59]. These findings mirror WHO Eastern Mediterranean Regional Office (EMRO) surveillance data showing rising carbapenem-resistant *Acinetobacter* and *Klebsiella* across North Africa and the Middle East. Persistent challenges, including limited isolation capacity, inconsistent stewardship and weak regional surveillance, sustain MDR transmission. Addressing these gaps is critical for improved patient survival and effective Antimicrobial Resistance (AMR) containment strategies.

## Conclusion

This study shows that MDR Gram-negative BSIs are highly prevalent in Libya and are associated with increased mortality, prolonged hospitalization and frequent inappropriate empiric therapy. The reduced efficacy of cephalosporins and carbapenems, alongside the limited but preserved activity of amikacin and colistin, highlights the urgent need for tailored empiric treatment, antimicrobial stewardship and infection control programmes to improve outcomes.

## Strengths, limitations and future directions

The study’s strengths include its prospective design, relatively large cohort and standardized phenotypic susceptibility testing, all of which enhance the reliability of the findings. Key limitations include the lack of molecular characterization of resistance genes (e.g. *NDM*, *OXA-48*), restriction to two tertiary hospitals and incomplete severity indicators such as Sequential Organ Failure Assessment (SOFA) scores and source-control data. Documentation gaps also limited analysis of empiric regimens, 30-day readmission and hospitalization costs, although the adequacy of empiric therapy was evaluated when possible. Fungal and mixed BSIs, as well as follow-up blood cultures, were not systematically assessed, potentially limiting interpretation of infection clearance. Despite these constraints, adjustment for major prognostic variables (ICU stay, comorbidity index, diabetes, empiric adequacy) supports the robustness of the results. Future research should integrate molecular epidemiology with clinical outcomes to clarify resistance transmission and inform regional stewardship, and also detecting carbapenemase genes (e.g. *NDM*, *OXA-48*) would strengthen understanding of MDR-GNB dissemination and support evidence-based policy development [60].

## Abbreviations

aOR: adjusted odds ratio
BSI: bloodstream infection
CI: confidence interval
MALDI-TOF: matrix-assisted laser desorption/ionization-time of flight
MDR: multidrug resistance
MDR-GNB: multidrug-resistant Gram-negative bacteria
